# Bioinspired lignocellulosic films to understand the mechanical properties of lignified plant cell walls at nanoscale

**DOI:** 10.1038/srep44065

**Published:** 2017-03-09

**Authors:** L. Muraille, V. Aguié-Béghin, B. Chabbert, M. Molinari

**Affiliations:** 1 FARE laboratory, INRA, Université de Reims Champagne-Ardenne, 51100, Reims, France; 2 Université de Reims-Champagne Ardenne, Laboratoire de Recherche en Nanosciences EA4682 F-51100 Reims, France

## Abstract

The physicochemical properties of plant fibres are determined by the fibre morphology and structural features of the cell wall, which is composed of three main layers that differ in chemical composition and architecture. This composition and hierarchical structure are responsible for many of the mechanical properties that are desirable for industrial applications. As interactions between the lignocellulosic polymers at the molecular level are the main factor governing the final cohesion and mechanical properties of plant fibres, atomic force microscopy (AFM) is well suited for the observation and measurement of their physical properties at nanoscale levels. Given the complexity of plant cell walls, we have developed a strategy based on lignocellulosic assemblies with increasing complexity to understand the influence of the different polymers on the nanomechanical properties. Measurements of the indentation moduli performed on one type of lignified cell wall compared with those performed on the corresponding lignocellulosic films clearly show the importance of the lignin in the mechanical properties of cell walls. Through this strategy, we envision a wide application of bioinspired systems in future studies of the physical properties of fibres.

There is a global demand for societies that are more environmentally friendly, relying less on fossil resources and more on renewable ones while protecting the environment. In this respect, lignocellulose biomass provides numerous opportunities for biorefineries to produce biomolecules, agromaterials or bioenergy^1^,^2^,^3^. Owing to their mechanical properties, low density and environmental benefits, plant fibres are already incorporated as reinforcements in many composites and the use of lignocellulosic polymers to produce sustainable material can benefit to the environmental impact of existing plant biomass industries. Indeed materials derived from plant fibres and related polymers, as mainly cellulose, lignin and hemicelluloses can offer several advantages, such as low cost, biodegradability, biocompatibility and renewability.

The physicochemical properties of lignocellulosic cell walls, such as their mechanical and hygroscopic properties, are attributed to their chemical composition as well as micro-and nanostructural features. Lignified cell walls are composites structures made of three main layers, showing chemical and structural gradient from the outer most layers, as primary wall and middle lamella to the inner most layer as secondary cell walls. The outer layers (middle lamella and primary wall) are the most lignified wall layers and mainly support intra-fibre cohesion in plant tissues. The secondary wall represents the main part of the cell wall and dictates the tensile properties of whole fibres and plant tissues. This layer can be summarized as a semicrystalline and orientated network of cellulose embedded in an amorphous matrix of hemicelluloses and lignin^4^. At molecular level, the cell wall network results from the formation of interactions between lignocellulosic polymers that involve both non-covalent linkages and covalent linkages^7^.

Understanding the effect of polymer interactions between individual polymers on the cell wall structure and subsequent impact on the cell wall properties is still challenging considering multi-scale analysis of the physicochemical properties of plant fibres from the nano to macroscopic level^8^,^9^,^10^. In this context, bioinspired systems can be a valuable tool because researchers can limit the number of polymers in the system or isolate the role of a specific parameter, such as the orientation of cellulose nanocrystals^11^, the mechanical and thermal properties of hemicelluloses^12^. The effect of the interactions between cell wall polymers on the properties of the corresponding bulk material have been mainly dedicated to the non-covalent associations of cellulose with hemicelluloses^12^ and lignin^13^. However most of the covalent interactions that provide cohesiveness to lignocellulosic cell walls involve non-cellulosic polysaccharides and lignin^14^. Moreover, distinct forms of lignin are present in wood fibre wall depending on the type of hemicellulose (glucomannan and xylan)^15^. Such covalent bonds have been reproduced by mimicking the lignification of the cell wall, i.e., by polymerizing the lignin monomer coniferyl alcohol in dilute solutions of pectins or arabinoxylans^16^,^17^. The final objective is a better understanding of the effect of each polymer and of their interactions (especially between polysaccharides and lignin) on the fibre properties. In addition, lignocellulosic networks can be a source of inspiration to design biobased materials with new potential applications in optical, packaging, medical, electronical fields^18^,^19^.

The goal, and novelty, of this study was the development of new lignified assemblies by the incorporation of lignin in cellulose- and hemicellulose-based films to address the influence of each cell wall polymer, notably lignin and their interactions on the spatial nanoheterogeneity of the topography and on the mechanical properties of the films. These measurements could allow the detection of a gradient of physicochemical and mechanical properties related to the nature and structure of polymers in the network with regards to measurements made on lignocellulosic cell walls. Specifically, we used atomic force microscopy (AFM), which allows for the investigation of not only morphological but also mechanical properties at nanoscale resolution on transverse sections of poplar stem and on nanostructured films. The latter were designed as non-lignified and lignified systems by modulating the interactions between the polymers. To lignify the films, a monolignol was mixed with the other polymers and then polymerized before preparing the films with the aim to mimic the lignification process in the plant cell wall to favour the covalent interaction between polysaccharides and lignin. This strategy allows changes in the supramolecular organization of the target material and in the local mechanical properties, as analysed by AFM, to be addressed.

## Results

### Mechanical properties of the fibre cell wall

**Figure 1:**
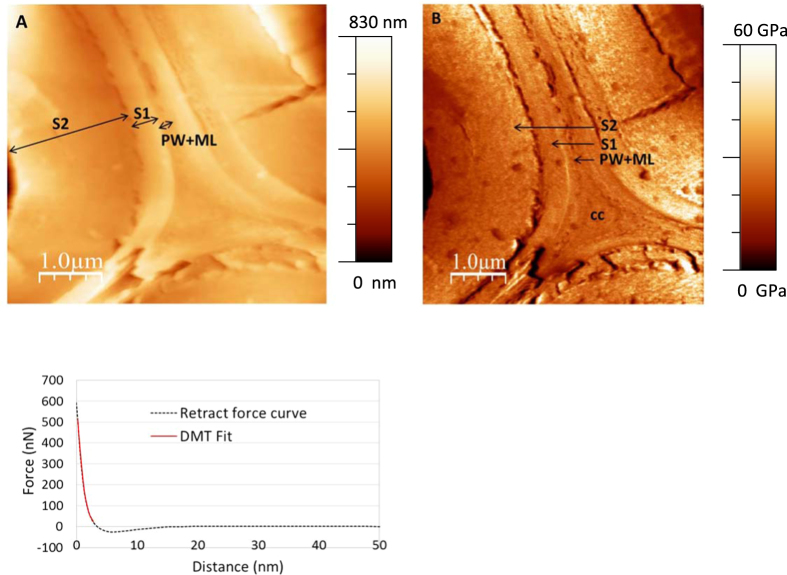
(**A**) AFM image of the topography of a poplar plant fibre cell wall. (**B**) Cartography of indentation modulus obtained by AFM (S1, S2: sublayers of the secondary walls, PW-ML: primary wall-middle lamella, cc: cell corner). (**C**) Typical force curve (S2 layer) with the associated DMT fit to extract the Indentation Modulus.

The topography of a poplar cell wall obtained by AFM is presented in Fig. 1A. The measurements were performed on a sample without any resin or glue between the sample and support to ensure that there was no interference with the modulus measurement. Additionally, the relative humidity was controlled at 40% to avoid variation in local mechanical properties due to water content^20^. The poplar sample contained almost 10% water according to the water sorption isotherm (data not shown).

**Figure 2:**
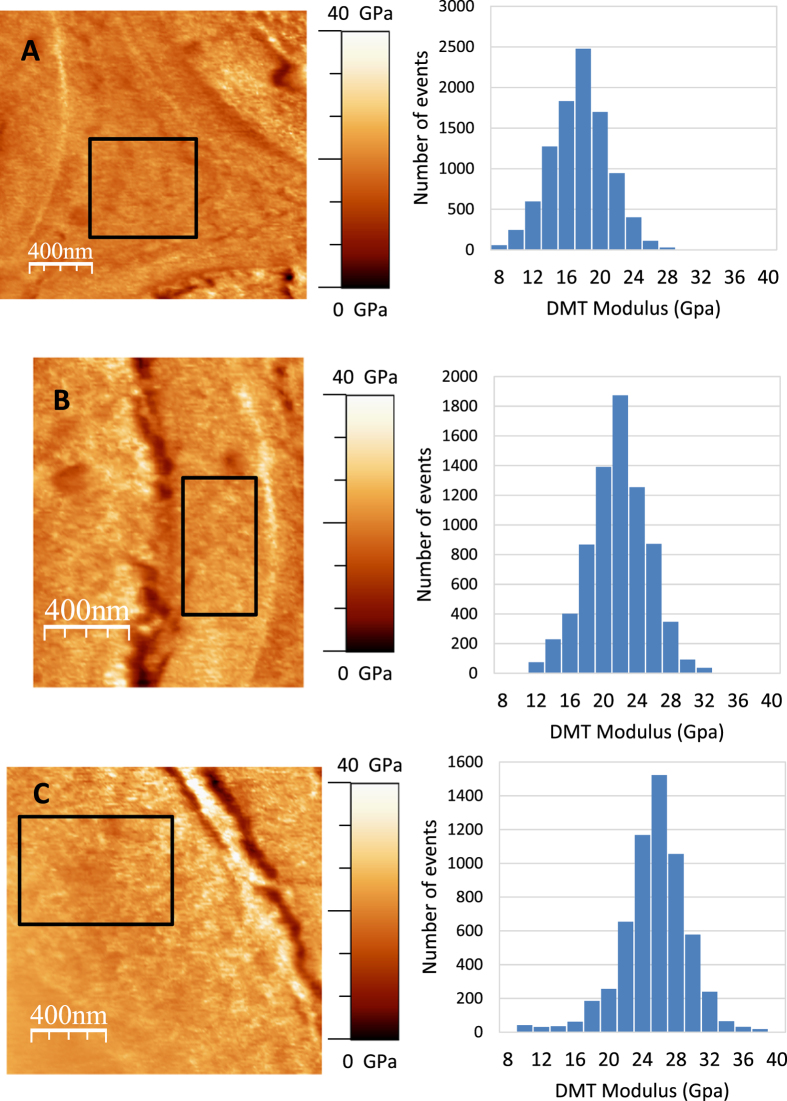
Indentation moduli obtained from the cell wall layers of poplar fibre. The black boxes indicate the areas where the indentation moduli were collected to obtain the histograms presented on the right. (**A**) Middle lamella/primary wall, (**B**) S1 layer, and (**C**) S2 layer.

The average thickness of the fibre cell wall is approximately 2.5 μm. The middle lamella/primary wall and the two secondary wall layers are readily visible and have thicknesses of 150 nm, 350 nm and 2 μm, respectively (Fig. 1A). Similarly, the indentation modulus cartography shows the presence of three layers (Fig. 1B), indicating a variation in mechanical properties between the cell wall layers (a magnified image of each layer is shown in Fig. 2). The histograms illustrating the occurrence of each indentation value calculated from the force-distance curve (Fig. 1C) and measured in the cell wall layers display a Gaussian profile (Fig. 2). The three histograms were obtained by collecting around 10000 measurements from each layer of the fibre cell wall for 5 samples prepared in similar conditions from the same poplar section. The indentation moduli of the middle lamella/primary wall area and the S1 and S2 layers averaged 17 ± 1.4 GPa, 21.1 ± 1.8 GPa and 25.9 ± 2.8 GPa, respectively (Fig. 2). The indentation modulus of the S2 layer, which represents 80% of the plant cell wall’s thickness, is in good agreement with the Young’s modulus obtained for spruce fibre (17 GPa)^21^ or for flax fibre (19 GPa)^22^. Our results indicate that the indentation moduli of the middle lamella/primary wall are lower than the corresponding values determined for secondary wall layers; other studies have also reported this disparity^23^. Moreover, the indentation modulus of the cell corner was observed to be similar to the indentation moduli of the middle lamella/primary wall (Fig. 2A).

The variations in the wall layers’ mechanical properties can be explained by the chemical and structural heterogeneity between the different layers, such as the variation in the content of cellulose and microfibril orientation along the cell axis^24^, in addition to the variation in the proportions of lignin and hemicellulose^23^,^25^,^26^. To observe how some of those variations affect the fibres mechanical properties, we designed and characterized nanostructured systems as thin films with varying contents of cellulose, lignin and hemicellulose. Before extending to films composed of several polymers, the nanomechanical properties of single-polymer films were first studied. These single-polymer films were then made progressively more complex to study the contribution of each polymer, notably lignin, and the polymers’ interactions on the final mechanical properties of the films.

### Mechanical properties of nanostructured films

#### Non-lignified films

**Figure 3:**
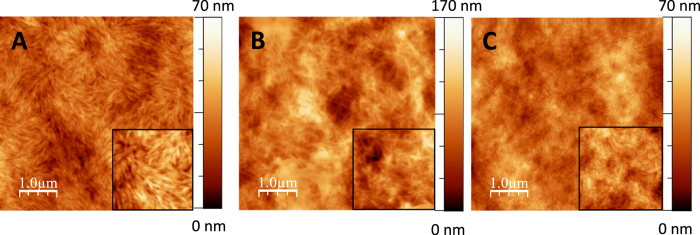
AFM images of single-polymer films. (**A**) CN film; (**B**) GM film; (**C**) XYL film. The insets on each image correspond to a zoom of 1.5 × 1.5 μm^2^.

Single-polymer films were prepared from cellulose nanocrystals (CNs), glucomannan (GM) and xylan (XYL) films; no cohesive films of synthetic lignin (dehydrogenopolymer of monolignol, DHP) could be obtained. All homopolymer films displayed a homogeneous surface on the nanometre scale (roughness measurement of the surface (Root Mean Square RMS) equal to 8, 20 and 7 nm for CN, GM and XYL films, respectively) (Fig. 3).

**Figure 4:**
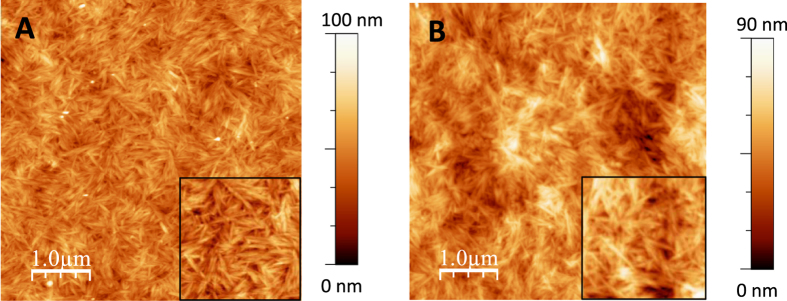
AFM images of non-lignified binary films. (**A**) CN/GM (4/1, w/w) and (**B**) CN/XYL (4/1, w/w). The insets on each image correspond to a zoom of 1.5 × 1.5 μm^2^.

As previously reported^27^,^28^,^29^, cellulose nanocrystals could be distinguished on the film surface with no particular orientation; however, the long axis of the nanocrystals appeared to be parallel to the film surface. AFM observation of the hemicellulose films revealed long filaments on the surface of the GM films. This pseudo-fibrillary form was previously observed by electron microscopy with recrystallized mannan II-type from dilute solution^30^. In contrast, this pattern was not observed for the XYL films, whose appearance was quite similar to that described in previous studies^31^. Binary films were designed using similar CN/hemicellulose ratios thereby allowing the comparison of xylan and glucomannan, which are the main hemicelluloses in secondary cell walls of hardwood and softwood respectively. We choose the ratio 4/1 so that the final ratio of ternary films 4/1/1 will roughly correspond to the proportion of cellulose/hemicellulose/lignin in wood fibres (50/25/25). The topographical images of non-lignified binary films indicate homogeneous composites with RMS values equal to 8 and 10 nm for CN/GM and CN/XYL, respectively (Fig. 4). Both binary films display a structure close to the single CN film suggesting that hemicelluloses were spread along the CNs due to tight, non-covalent interaction. Accordingly, several studies have indicated that hemicelluloses can adsorb to the surface of cellulose^32^. Hence, it is suggested that this binding occurs preferentially with structures such as hemicellulose containing β-(1→ 4) glucosyl linkages and a conformation that is complementary to cellulose.

**Figure 5:**
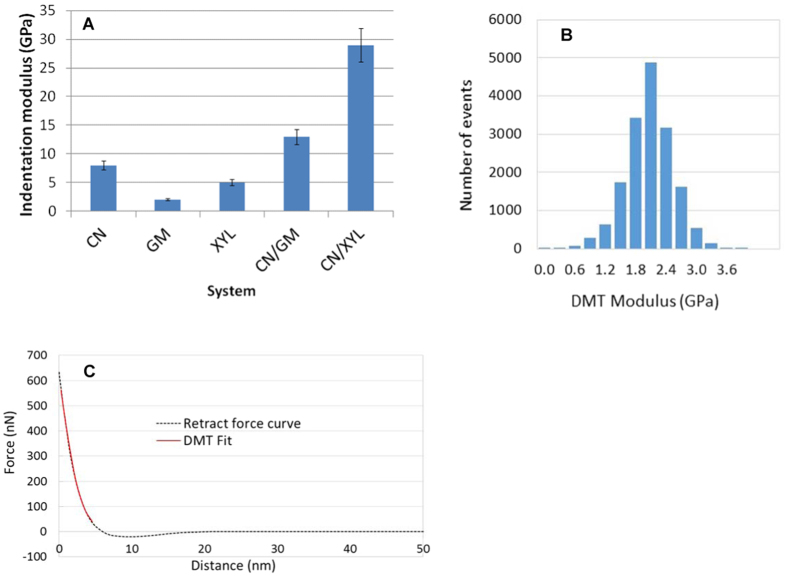
Mechanical properties of the non-lignified single-polymer and binary films. (**A**) Indentation modulus of non-lignified films; (**B**) Gaussian distribution of the indentation moduli determined for GM films; (**C**) Typical force curve (CN/GM sample) with the associated DMT fit to extract the Indentation Modulus.

The indentation moduli determined for the non-lignified films are reported as the frequency of each value of the indentation modulus (Fig. 5A). In all cases, the values deduced from the force-distance curves (Fig. 5C) follow a pure Gaussian distribution, which indicates that the formation is homogeneous with a uniform distribution of the polymers at the surface of the films, as shown in the topographical images (Fig. 5B). The moduli were observed to be equal to 7.8 ± 0.8, 1.9 ± 0.2 and 5 ± 0.4 GPa for the CN, GM and XYL films, respectively. They are positively related to the respective density measured at dry state respectively: 1.547 ± 0.009; 1.480 ± 0.006 and 1.515 ± 0.008.

For the CN films, our results are consistent with the horizontal position of the nanocrystals shown in the AFM images (see insets in the Figs 3 and 4 for instance); thus, the tip applied a force perpendicular to the axis of the nanocrystals. The value observed for cellulose is in good agreement with the transversal moduli data reported in several studies involving nanoindentation (8.3 GPa^33^). However, other studies have reported transversal indentation moduli varying between 11 and 50 GPa^27^,^34^. These differences can be explained by several factors, including the crystal structure of the nanocrystals, degree of crystallinity, anisotropy, defects, relative humidity, density of film during the measurements and the differences in the experimental techniques used. For example, wood CNs exhibit a higher degree of crystallinity (77%) than ramie CNs (56% in our case), but the two types are similar in diameter (5 nm); nonetheless, the estimated indentation modulus reached almost 20 GPa for both CNs. Moreover, the contact model used to calculate the indentation modulus strongly affects the values obtained. For example, the transverse elastic modulus of cotton CNs is calculated to be 5 and 18 GPa using the Hertz model and finite element analysis, respectively^27^. Consequently, it must be noted that these values are not necessarily absolute but can be used to compare the mechanical properties of different films. Nevertheless, it is interesting to note that indentation moduli in the same range, *ie* 8 GPa, were obtained for cellulose films using nanoindentation (Triboscope, Hysitron Inc., Eden Prairie, MN; using a Berkovich indenter with a tip radius of approximately 300 nm, data not shown).

The indentation moduli obtained for the GM films (1.9 ± 0.2 GPa) were within the same range of values as those obtained by dynamic mechanical analysis (DMA) performed on similar films (approximately 2 GPa^12^) but differed slightly from the results obtained by indentation measurements performed on powder glucomannan tablets on the macroscopic scale (approximately 8 GPa at 40% RH^35^). However, the latter measurements were performed on a rod composed of compressed hemicellulose (glucomannan or xylan) powders, which was greatly different from the films used in our study. The average indentation modulus of the XYL films was 5 GPa, slightly higher than the values determined for the GM films (2 GPa). This difference cannot be entirely explained by the low difference in water content (1%) between the two XYL and GM films (Table 1), but may be the result of the different patterns formed by the polymers during film formation, as observed by AFM imaging.

Similarly, the high proportion of crystalline cellulose (60%) and lower water content (5%) of the CN films compared to the XYL and GM films (11% and 12%, respectively) (table 1) may explain why the indentation modulus of the CN films was higher than that of the amorphous hemicellulose films. The average indentation moduli of the binary films were 15.2 ± 1.5 and 29 ± 2.9 GPa for the CN/GM and CN/XYL films (4/1), respectively. Therefore, the indentation moduli of the binary films were higher, being 90 and 260% greater for the CN/GM and CN/XYL films, respectively, than for the pure cellulose films. This increase cannot be explained by the water content (table 1) or by the density which values are similar (1.560 ± 0.008 for CN/GM films and 1.550 ± 0.004 for CN/XYL films). The value obtained for the CN/GM films was higher than the values obtained for systems containing less cellulose (for 15% cellulose, 2 GPa as determined by DMA^12^). This result suggests that the films were not simply a mixture of the two polymers but a novel structured system involving non-covalent interactions between the surface of the cellulose nanocrystals and hemicelluloses, which led to a significant increase in the indentation modulus. This observation may be explained by the non-covalent interactions between cellulose and hemicelluloses identified in previous studies by FTIR in native systems (spruce wood)^7^. In addition, adding a small amount of arabinoxylan to pure cellulose films has been shown to induce higher indentation moduli (6.6 and 7.3 GPa for pure nanofibril cellulose films and films containing 25% arabinoxylan and 75% cellulose, respectively)^36^. The weaker increase of modulus reported in the latter study can be related to the morphology of cellulose and the structure of xylan. Nanowhiskers display higher accessible surface for xylan adsorption as compared to nanofibril. Moreover, less substituted xylan, ie lower proportion of arabinose on the xylan backbone is more prone to interact with cellulose^37^, (Ara/Xyl ratio = 0.23, this study^38^ instead of 0.50 in Stevanic *et al*.^36^). Similarly the variation in the mechanical properties of the CN/GM and CN/XYL films can be explained by the extent of non-covalent interactions formed between the two polysaccharides and cellulose.

To conclude the study of the non-lignified films, although observed in a lower proportion than cellulose, both hemicelluloses appeared to exhibit a sufficient level of interaction with cellulose to produce homogeneous films on the macroscale and nanoscale. Moreover, these interactions allowed for the enhancement of the films’ mechanical properties, which were observed to be higher than those obtained by single-polymer films. The next step of this study was the introduction of lignin into the polymer systems by polymerization of monolignol (coniferyl alcohol) with peroxidase and hydrogen peroxide in presence of polysaccharide^38^.

#### Lignified films

Lignified films were produced to study the effect of lignin and that of interactions with polysaccharides on the mechanical properties of the films. The polymer weight ratios were 1/1 for hemicellulose/lignin films, 4/1 for cellulose/lignin films and 4/1/1 for cellulose/hemicellulose/lignin films. These polymer ratios were first selected to mimic the large chemical variation of the plant secondary cell walls; for instance, wood fibres contain highly cellulosic cell walls (~60% cellulose) with ~20% hemicelluloses and ~20% lignins 1. In plant cell walls, the polymerization of monolignol occurs in the pre-existing matrix of polysaccharides and can lead to the formation of strong and covalent linkages with hemicellulose^39^. Therefore, to promote more interactions between the polymers, model lignin, DHP was polymerized in the presence of the other polymers in aqueous medium without an organic solvent. This polymerization allowed for the formation of homogeneous films on the macroscopic scale with no phase separation^38^.

**Figure 6:**
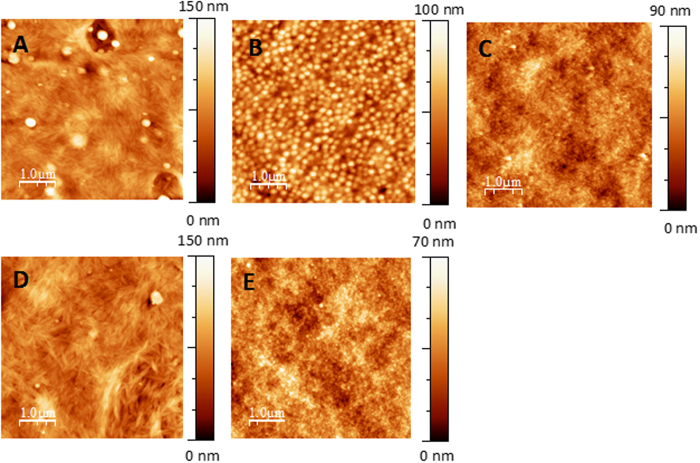
AFM images of lignified films. (**A**) CN/DHP (4/1, w/w), (**B**) GM/DHP (1/1, w/w), (**C**) XYL/DHP (1/1, w/w), (**D**) CN/GM/DHP (4/1/1, w/w/w), and (**E**) CN/XYL/DHP (4/1/1, w/w/w) films.

AFM images of these systems are shown in Fig. 6. The surface of each film exhibited nodules, which may be mainly attributed to the lignins incorporated into the films, as previously reported^19^. The nodule density (average size) was 2 nod/μm^2^ (average size of 240 nm), 30 nod/μm^2^ (160 nm), 800 nod/μm^2^ (35 nm), less than 1 nod/μm^2^ (360 nm) and 200 nod/μm^2^ (52 nm) for the CN/DHP, GM/DHP, XYL/DHP, CN/GM/DHP and CN/XYL/DHP films, respectively. Thus, the size of the nodules varied with the type of polysaccharides incorporated into the films: smaller but more numerous nodules occurred in the xylan-based ternary films relative to the glucomannan-based ternary films. This pattern may have resulted from the variation in monolignol’s affinity towards the different hemicelluloses^15^. In our study, the lignin produced *in vitro* had better affinity with xylan. Moreover, nodules were observed to be smaller in the presence of xylan than in the presence of glucomannan and cellulose. Previous studies have shown that lignin can improve the affinity between xylan and cellulose^40^,^41^. Moreover, lignin–hemicellulose complexes exhibiting covalent interactions have been shown to enhance lignin dispersion^42^. Consequently, higher affinities between xylan and lignin and between xylan and cellulose allowed for the formation of the most nanoscopically homogeneous films among all the lignified films.

**Figure 7:**
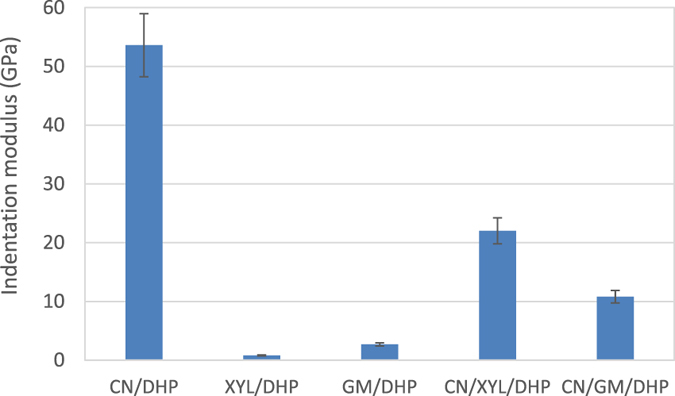
Indentation moduli of lignified films. The ratio of each sample is the same as that reported in Fig. 6.

The indentation moduli of the lignified systems are presented in Fig. 7. These films showed a wide range of indentation moduli: except binary CN/DHP film, the hemicellulose/DHP systems exhibited weak values of indentation moduli: 0.22 ± 0.02 GPa for the XYL/DHP and 2.7 GPa for GM/DHP films. The incorporation of crystalline cellulose in ternary films contributed to a significant increase of the indentation modulus up to 10.8 GPa for CN/GM/DHP and 22 GPa for CN/XYL/DHP films. These differences can be related to the difference of i/ AFM topographical images suggesting that during the growth of DHP, more covalent bonds are formed with xylan than with any other system and ii/ the water content in the film at 40% RH, which is higher for the XYL/DHP than for the GM/DHP films (Table 1). This water could act as a plasticizing agent, which—in combination with the high affinity between xylan and DHP—would lead to novel mechanical properties.

**Figure 8:**
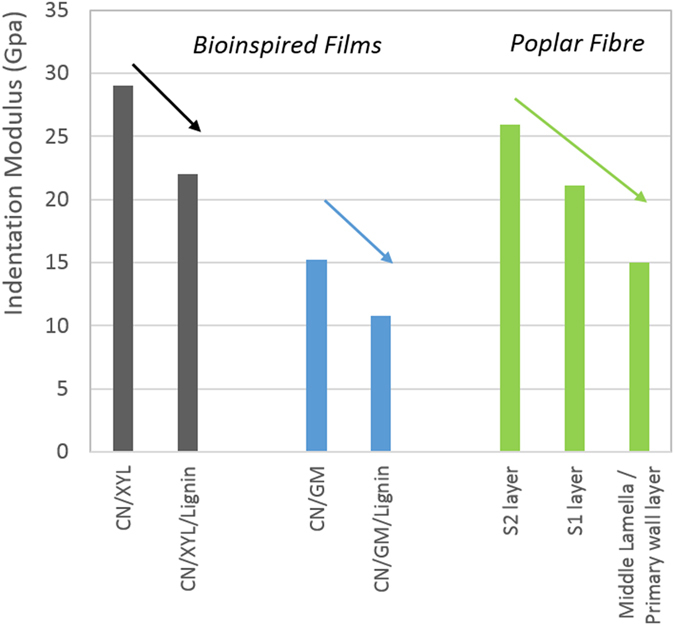
Lignin impact on the indentation moduli of reconstituted films in comparison with the indentation moduli of the different layers of the poplar fibre.

A comparison of the results obtained for the films with the measurements performed on poplar fibres indicates that the indentation moduli of ternary systems are in the same range as the values obtained for the different layers of the fibre cell wall (11 and 22 GPa for CN/GM/DHP films and CN/XYL/DHP films, respectively, compared to 15–25 GPa measured for the cell wall of poplar fibres). With respect to the reconstituted systems, mixing the hemicelluloses with cellulose led to the formation of films with enhanced mechanical properties compared with those of the single-polymer films. Except for the CN/DHP systems, all lignified films exhibited a decrease in their mechanical properties when compared with those of the same systems without any lignin as seen in Fig. 8 (for example, the indentation modulus of CN/XYL/DHP was 22 GPa, while that of the CN/XYL films was 28 GPa). Consequently, the presence of lignin led to a decrease in the mechanical properties of the films related to the decrease of cellulose content in lignified films (80% cellulose in CN/DHP films compared to 66% CN in ternary films and 0% CN in hemicellulose/DHP films). This observation is consistent with another study performed on woody hemp fibre that showed that different fibres with decreasing concentrations of lignin presented increasing mechanical properties, even when the concentration was varied by a very small degree (the mechanical properties increased by 37% when the concentration of lignin was reduced by 1.5%^43^). Therefore, although the cellulose nanocrystals in our systems were not oriented, they appeared to have adequately reproduced the decrease in mechanical properties observed when the polysaccharide network was lignified. Concerning the measurements performed on the fibres, the S2 layer was observed to have a higher indentation modulus than the S1 layer, which in turn showed a higher indentation modulus than the middle lamella/primary wall. Based on our results, we can conclude that the decrease in the concentration of lignin and the parallel increase in the concentration of cellulose from the middle lamella/primary wall to the S2 layer are partly responsible for the increase in the indentation modulus. It should be recalled, however, that the enhancement in the orientation of the cellulose nanofibrils from S1 to S2 also contributes to this measurement and was not studied in this work.

## Conclusions

The nanomechanical properties of cell wall polymers were measured on the cell wall layers of poplar fibres and on thin lignocellulosic films. Using the three main plant cell wall polymers (cellulose, hemicellulose and lignin), reconstituted films were designed to mimic some of the structural features rather than the complete cell wall architecture. Notably, increases in film complexity and modulation of the interactions between polymers were achieved.

Thus, the indentation moduli of the mixed cellulose/hemicellulose films were higher than those of the pure polymer films, indicating the formation of interactions between the polysaccharides during film preparation. The polymerization of lignin monomer in the presence of the polysaccharides mimics the mechanism of lignin formation in plant cell walls and promotes the formation of numerous bonds between the polymers and/or a higher affinity between polymers and the production of homogeneous films with novel properties compared with those observed for single- or mixed-polymer films^38^. Thus, depending on the type of polysaccharides used, films with varying mechanical behaviour could be obtained, from hydrogel-like xylan/DHP films to very stiff CN/DHP films. The mechanical properties of the films (from 0.22 to 54 GPa) cover a wider range than those determined at the nanometric scale on different cell wall layers of a poplar sample. Moreover, the indentation modulus increases gradually from the lignin-rich middle lamella/primary wall layer to the secondary wall layers that differ in cellulose and lignin concentration (from approximately 17 to 26 GPa), as in nanostructured films following progressive complexification of the polysaccharides and lignified network (from 0.22 to 22 GPa with xylan and from 2.7 to 11 GPa with glucomannan). Moreover, properties of these cellulose based systems may reveal different nanomechanical properties according to the structure of hemicellulose and its affinity with lignin. Studies on bioinspired films using various polymer ratios and other non-cellulosic polysaccharides could provide further insights into the nanomechanical properties of the plant cell wall which composition varies in content and structure of lignin and non-cellulosic polysaccharides. Cell wall polymer films may serve as reference systems for studies extending to different cell types and species.

## Materials and Methods

### Plant Material

Woody samples were obtained from stems of one-year-old poplar *(Populus tremula x Populus alba*, INRA clone 717–1B4) grown in a greenhouse. Small stem portions (5 × 2 × 2 mm thick) were collected 20 cm above ground level prior to fixation in 70% ethanol. Then, the samples were dehydrated in an ethanol series prior to resin impregnation and embedding using methyl- and butyl-(1:1, v/v) methacrylate resin (Sigma-Aldrich, Saint-Louis, USA). For AFM nanomechanical measurements, 1-μm transverse sections were obtained from embedded specimens using a microtome (MICROM, Francheville, France) and placed onto an AFM disk; the resin was then removed using acetone as previously described^44^.

### Polysaccharides and lignin polymers

Glucomannan and xylan solutions were obtained from Konjac glucomannan (Megazyme, Wicklow, Ireland) and a water-soluble xylan purified from oat spelt^45^, respectively. Hemicellulose solutions were prepared as described by Muraille *et al*.^38^. A lignin model compound (dehydrogenation polymer, DHP) was synthesized by the slow and continuous addition of coniferyl alcohol (4-hydroxy-3-methoxy-cinnamyl alcohol) and hydrogen peroxide to an aqueous solution of peroxidase according to the procedure detailed in ref. 46. Based on the system elaborated, cellulose and/or hemicelluloses can be added to the solution before initiating the polymerization of the alcohol^38^, as recently used by^47^.

### Formulations and film preparations

The composition of the films and the manner in which the films were prepared were described previously^38^. Briefly, in addition to single-polymer films (CN, GM and XYL for cellulose, glucomannan and xylan, respectively), binary systems containing cellulose/hemicellulose (4/1 w/w) or polysaccharides/DHP (1/1 w/w) were prepared. Finally, ternary films containing cellulose, hemicelluloses and lignin (4/1/1 w/w) were prepared by introducing lignin by coniferyl alcohol polymerization in the presence of the polysaccharides. Density was determined by the buoyancy method using Argon gas with IGA System according to the supplier (Hiden Isochema, Warrington). Average film thickness, measured with a dual-thickness gauge (precision of ± 0.001 mm) (Käfer GmbH, Villingen, Germany), was 30 ± 5 μm.

### Surface morphology and nanomechanical measurements by atomic force microscopy

Surface topography and elastic moduli measurements were performed on a Multimode 8 AFM in Peakforce QNM mode (Bruker, USA) with the 9.10 version of the Nanoscope Software. All measurements were performed at room temperature and at a controlled relative humidity of 40%. The water content of the films at 40% RH was measured using a gravimetric sorption analyser^38^. Quantitative measurements were performed using tips that cover the range of mechanical properties exhibited by the samples^48^. Indeed, the tip must be sufficiently stiff to deform the sample while retaining enough sensitivity to measure the indentation modulus. Consequently, five tips with nominal spring constants of 0.4, 5, 40, 200 and 350 N/m were used. The deflection sensitivity was determined using a clear sapphire sample and is an average of 3 different measurements at the beginning and recalculated several times during the measurements. The spring constants of the cantilever were estimated using the thermal tuning method and Sader method for values above and below 1 N/m, respectively. The apex radius of the AFM tips (between 20 and 40 nm depending on the tip) was systematically determined using reference samples (Bruker, USA) with known Young’s moduli with a controlled deformation depth of 5 nm. This depth was chosen because the deformation must be less than one-tenth of the film’s thickness to avoid substrate effects and sufficiently low to remain within the elastic regime of the sample. The radius was checked before and after measurements. If differences were noticed, the force curves were reprocessed in order to take this change in radius into account. Special care was taken to subtract the background noise and synchronize the approach and retraction curves. The applied force for the nanomechanical measurements was of 600nN. For each sample, the topography and elastic modulus were collected from three different plant cell walls of the same tissue. For each plant cell wall, up to ten different locations were imaged over areas of 5 × 5 μm at a digital resolution of 512 × 512 pixels (for a total of more than 262 000 collected curves and corresponding indentation moduli per image). The vertical oscillation frequency of the probe was 1 kHz.

To extract the elastic modulus from the force-distance curves, an appropriate contact model is required depending on the geometry of the contact between the sample and the tip. Three models are mainly used for flat and stiff samples: the Hertz^49^, DMT^50^, and JKR^51^ models. In contrast to the two other models, the Hertz model does not take adhesion forces into account. Because the tips used in this study are quite sharp, the DMT model is more appropriate because this model takes into account the forces acting outside the contact area, which are not negligible for tips with a sharp apex^52^. The elastic modulus was deduced from force-distance curves using the DMT model for a spherical indenter^53^ via the equation 1:


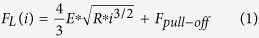


where *F*_*L*_ is the applied force, E* is the reduced modulus (or indentation modulus), R* is the reduced radius 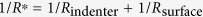. *i* is the indentation depth and *F*_*pull-off*_ is the force at the point of pull-off of the AFM probe from the surface. Given that the mechanical measurements were only performed on flat surfaces, the reduced radius could be simplified as the radius of the indenter.

## Additional Information

**How to cite this article:** Muraille, L. *et al*. Bioinspired lignocellulosic films to understand the mechanical properties of lignified plant cell walls at nanoscale. *Sci. Rep.* **7**, 44065; doi: 10.1038/srep44065 (2017).

**Publisher's note:** Springer Nature remains neutral with regard to jurisdictional claims in published maps and institutional affiliations.

## Figures and Tables

**Table 1:** Water content of the lignocellulosic films at 40% RH.

Systems	Water content (in % w/w)
CN	4.7
GM	12.1
XYL	10.8
CN/GM	6.2
CN/XYL	4.6
CN/DHP	4.7
GM/DHP	9.5
XYL/DHP	12.7
CN/GM/DHP	5.1
CN/XYL/DHP	6.1
